# Epigenetic mechanisms shape the underlining expression regulatory mechanisms of the STAT3 in multiple sclerosis disease

**DOI:** 10.1186/s13104-020-05427-1

**Published:** 2020-12-29

**Authors:** Arezoo Hosseini, Zohreh Babaloo, Tohid Gharibi, Navid Shomali, Faroogh Marofi, Vida Hashemi, Hormoz Ayromlou, Milad Asadi, Shima Rahmani, Saeed Noorolyai, Dariush Shanehbandi, Behzad Baradaran

**Affiliations:** 1 Student Research Committee, Tabriz University of Medical Sciences, Tabriz, Iran; 2 Immunology Research Center, Tabriz University of Medical Sciences, Tabriz, Iran; 3 Department of Immunology, School of Medicine, Tabriz University of Medical Sciences, Tabriz, Iran; 4 Aging Research Institute, Tabriz University of Medical Sciences, Tabriz, Iran; 5 Department of Neurology, Tabriz University of Medical Sciences, Tabriz, Iran; 6 Department of Basic Science, Faculty of Medicine, Maragheh University of Medical Sciences, Maragheh, Iran

**Keywords:** STAT3, Multiple sclerosis, Methylation, Iran

## Abstract

**Objectives:**

Immunological tolerance is mediated by CD4^+^CD25^+^ regulatory T (Treg) cells. Studies have shown that thymic and peripheral generations of Treg cells depend on the CD28 signaling pathway. T helper 17 (Th17) cells are involved in the pathophysiology of various inflammatory diseases. Cytokines, such as interleukin (IL)-6 and TGF-β, regulate the reciprocal development of Th17 and Treg cells. In CD4^+^ T cells, signal transducer and activator of transcription 3 (STAT3) play a critical role in the induction of Th17 cell differentiation and inhibition of Treg cell development.

**Results:**

In this study, we investigated the STAT3 methylation and gene expression status in patients with MS. Our study demonstrated that the level of STAT3 methylation decreased in relapsing–remitting MS patient compared to control groups, which the decreases were statistically significant. STAT3 gene expression increased in patient group relative to healthy one, and the increases were found to be statistically significant. According to our findings, it can be suggested that DNA hypermethylation of STAT3 affects the gene expression. In addition, there is a strong and significant negative correlation between the methylation status and mRNA level of STAT3.

## Introduction

Multiple sclerosis (MS), a chronic inflammatory disease of the central nervous system (CNS), has been evidenced to cause demyelination and axonal degeneration within the brain and spinal cord [1, 2]. The exact etiopathology of MS has not yet been clarified, but most studies have recognized MS as an autoimmune disease mediated by autoreactive CD4^+^ T cells [3].

Immunological tolerance is a critical factor in the prevention of chronic infection, cancer, and autoimmune diseases [4]. Central tolerance operates in the thymus where autoreactive T cells with high affinity for self-antigens are negatively deleted [5]. Given that not all antigens are present in the thymus, self-reactive T cells can enter the peripheral blood [5]. Therefore, central tolerance alone is insufficient, and peripheral tolerance mechanisms are required [6]. CD4^+^CD25^+^ regulatory T (Treg) cells are major suppressor T lymphocytes and mediate peripheral tolerance [7, 8]. Forkhead box P3 (FOXP3) transcription factor is also necessary for the differentiation of Treg cells [9, 10]. Although Treg cells differentiate naturally in the thymus, these cells can also be generated from CD4^+^CD25^+^ naive T cells into adaptive Tregs in the periphery [11–13]. Adaptive Treg cells can be induced in the periphery when encountered with repeated antigens [14].

Researchers have suggested that the CD28/B7 costimulatory molecule is essential for the expression of CD25 and FOXP3 on Tregs [15–17]. It has also been indicated that in the absence of the CD28 costimulatory pathway, the peripheral number of Tregs decreases [16]. Besides, Lck-binding motif in the cytosolic tail of CD28 is required for Tregs generation [18]. However, it is uncertain how CD28 leads to the FOXP3 expression and Treg development [16]. Treg cells are involved in maintaining anergic state and exert suppressive function in various inflammation and autoimmune diseases, including rheumatoid arthritis, systemic lupus erythematosus, and MS [17, 19–21].

In MS patients, autoreactive CD4^+^ T cells display mainly T helper 17 (Th17) phenotype [3]. Cytokines, such as TGF-β and interleukin (IL)-6, play a key role in regulating Th17 cell differentiation [3, 22]. Retinoic acid receptor-related orphan receptor (ROR) γt transcription factor induces the differentiation of naive CD4^+^ T cells into Th17 cells [22, 23], which are pathogenic in MS due to the production of cytokines such as IL-17, IL-21, and IL-22 [24]. In MS, IL-17 leads to blood–brain barrier disruption and clinical disease activity and symptoms [25]. The upregulation of RORγt is dependent on STAT3 [26]. Following the binding of IL-6 to IL-6R, STAT3 is phosphorylated on Tyr^705^, dimerizes, moves into the nucleus and regulates the gene expression [27, 28]. STAT3 functions distinctly in the Th17 development and regulation of the Th17/Treg balance [23], and STAT3 deficiency impairs RORɣt expression, giving rise to the increased expression of FOXP3 [29]. Therefore, dysregulation of STAT3 results in the development of various inflammatory diseases, and loss of STAT3 in naïve CD4^+^ T cells inhibits the development of CNS inflammatory diseases [30, 31]. Several studies have introduced STAT3 as a risk factor allele for MS disease susceptibility [32–34]. These observations persuaded us to investigate whether the hypo- or hyper-methylation of STAT3 in CD4^+^ T cells is associated with the susceptibility of MS.

In this study, we display that in CD4^+^ T cells, STAT3 methylation decreases in relapsing–remitting MS (RRMS), whereas the gene expression of STAT3 increases.

## Main text

### Methods

#### Study groups

A total of 50 MS patients (36 males and 14 females) aged between 19 and 65 years with clinically RRMS were collected from the Imam Reza Hospital of Tabriz University of Medical Sciences, East Azerbaijan Province, Iran. All the patients had RRMS according to the McDonald's diagnostic criteria and were in the remission clinical phase. Disease remission was defined as improvement from baseline clinical status for at least three months. The cases were weekly being treated with interferon beta. Normal controls enrolled in this study were composed of 50 age, gender, and ethnically matched healthy subjects without any clinical or laboratory signs of autoimmune or inflammatory diseases. A written informed consent was obtained from each case, and the study protocol was approved by the Ethics Committee of the Tabriz University of Medical Sciences. The clinical/pathological data of both RRMS and controls are summarized in Table 1.

**Table 1:** Clinical characteristics of RRMS and control subjects

Characteristics	RRMS group (n = 50)	Control group (n = 50)
Age	35.08 (19–65)	33.01 (22–51)
Gender (female/male)	36/14	31/19
EDSS	1.75 ± 0.31	NA
Disease duration	4.9 ± 1.6 (2–15 years)	NA

Data are shown as mean ± SD or frequencies

*NA* non-applicable, *EDSS* expanded disability status scale

#### Blood sampling and cell isolation

Peripheral blood samples (20 ml) were obtained from all the patients with RRMS. After the blood collection, peripheral blood mononuclear cells were isolated using Ficoll-Paque™plus gradient centrifugation (Biosera, UK) within 12 h. The isolation of CD4^+^ T cells from peripheral blood mononuclear cells was carried out with the Miltenyi Biotech’s MACS System. The CD4^+^ MACS Isolation Kit was applied to positively select CD4^+^ T cells. The purity of the CD4^+^ T cells was assessed with flow cytometry and assigned to be greater than 90%.

#### DNA extraction and methylation-specific quantitative polymerase chain reaction (MS-qPCR)

Total DNA isolated from the CD4^+^ T cells was gathered in EDTA-containing tubes by the salting-out method. STAT3 promoter sequences and data were obtained from the NCBI (National Center for Biotechnology Information) database. STAT3 expression primers were designed by the aid of the PrimerQuest Tool, and the methylation- and demethylation (DM)-specific primers for STAT3 were designed using MethPrimer online database and OLIGO software. The primer sequences and product size for STAT3 are shown in Table 2. The methylation status of STAT3 was analyzed by applying the MS‐qPCR. Power SYBR Green reagent (Thermo Fisher Scientific, USA) was utilized for MS-qPCR. The DM rate of STAT3 was calculated by a previously described formula [35, 36] in which DM is the ratio of amplification efficiency of the methylated to unmethylated samples:

$$ {\text{DM}}\%  = 100/[1 + 2({\text{Ct.TG}} - {\text{Ct.CG}})]. $$

**Table 2:** PCR primers, melting temperature, and product size

Primers		Sequence	Melting temperature	Product Sizes (bp)
STAT3	MF	TATCGTTTTTTGTATTCGTTTGTAC	58.2	192
	MR	CCTACTTTAAACTTCAATTTCTACGTA	59.0	
	UMF	TTGTTTTTTGTATTTGTTTGTATGG	57.5	
	UMR	CCTACTTTAAACTTCAATTTCTACATA	57.5	190

#### RNA isolation and reverse transcriptase (RT)-PCR

Total RNA from the collected CD4^+^ T cells was isolated using TRIzol Reagent (Life Technologies, USA) based on the manufacturer’s instructions. RNA was then reverse transcribed with the Prime Script™ RT reagent Kit (Takara, Japan) as per the protocol recommended by manufacturer. Subsequently, SYBR Green reagent (Thermo Fisher Scientific) was used for quatitative real‐time (qRT‐PCR). Pfaffl method [37] was applied to calculate the relative gene expression. PCR cycles included a holding cycle at 95 °C for 15 min and held at 80 °C before the addition of 1.25 units of Taq polymerase (Invitrogen, USA). The forward and reverse primers used for STAT3 expression were comprised of 5′-TGGAGCTGCGGCAGTTTCTG-3′ and 5′-CCGCATCTGGTCCAGCGCAG-3′, respectively [38]. For STAT3, the temperature condition was as follows: 30 cycles of 95 °C for 1 min, 63 °C for 1 min, followed by one cycle of 72 °C for 5 min. The mRNA expression level was normalized against glyceraldehyde-3-phosphate dehydrogenase (GAPDH) mRNA. STAT3 primer sequences are listed in Table 2.

#### Statistical analysis

Statistical analysis was performed with SPSS software version 25 (IBM Corp., Armonk, NY, USA). All the data were presented as mean ± standard error of mean (SEM). Kolmogorov–Smirnov test with P–P plot and Q–Q plot was employed for normal distributions. The differences in the mRNA level of STAT3 between RRMS patient and control groups were evaluated by unpaired t-test. A P-value < 0.05 was considered as statistically significant difference.

### Results

#### STAT3 expression in the study groups

**Fig. 1:**
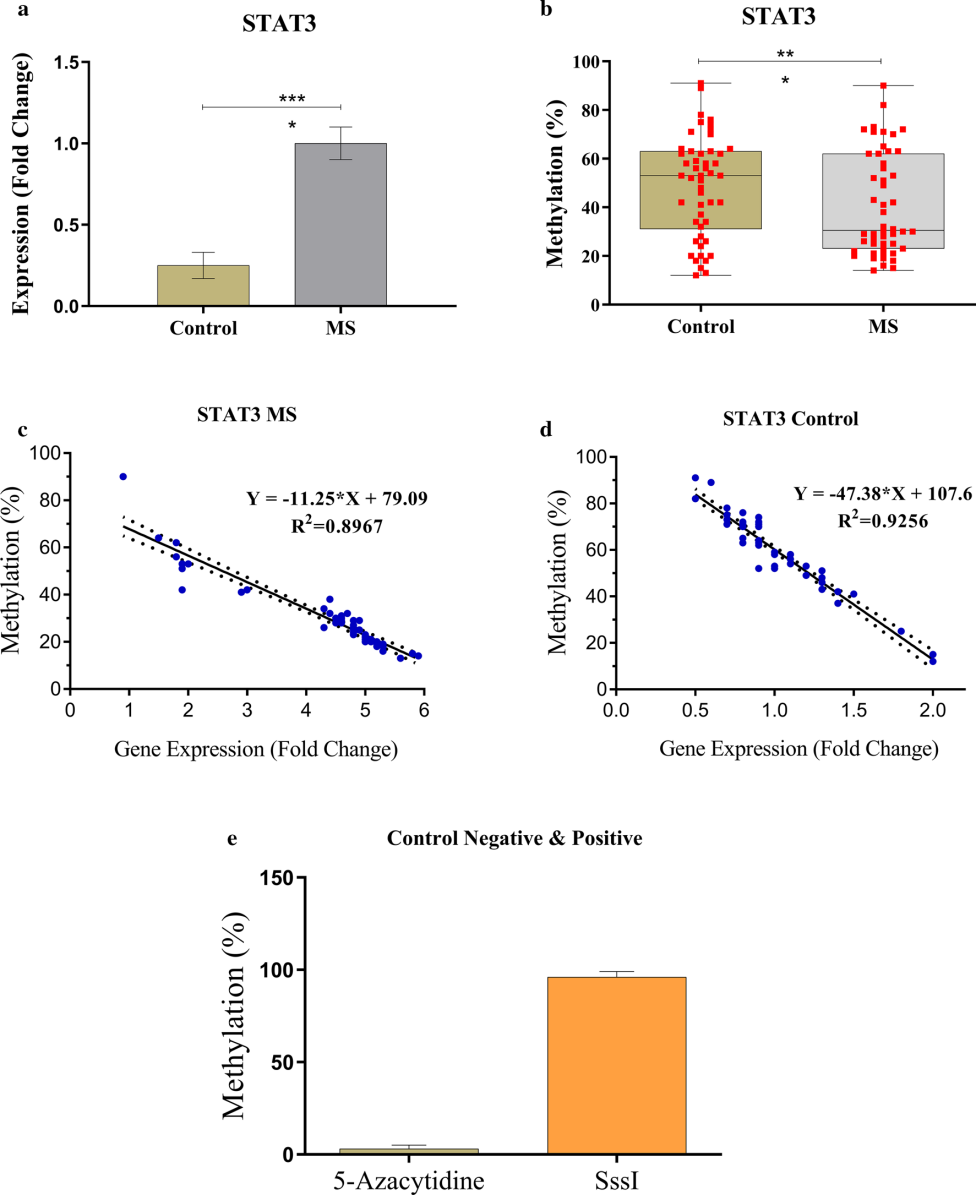
The gene expression and promoter methylation level of the STAT3 gene in MS and control groups. **a** STAT3 gene expression in patients compared with control subjects. Mean fold change in MS patients and control group was 1.000 ± 0.024 and 0.250 ± 0.014, respectively (P-value < 0.0001). **b** The methylation level (% M) of STAT3 in MS patients compared with control group. The red dots are individual values, and boxes are the mean methylation for patient (30.56 ± 1.317) and control groups (59.08 ± 1.986). **c**, **d** Correlation analysis between methylation status and gene expression level of STAT3 in patients (R2 = 0.8967, P-value < 0.0001, regression analysis) and control (R2 = 0.9256, P-value < 0.0001, regression analysis) group. **e** Negative and positive controls for methylation assays. 5-Azacytidine and DNA sample treated with SssI methyltransferase were used as negative and positive controls, respectively [39]. (*P-value < 0.05, **P-value < 0.01, ***P-value < 0.001, ****P-value < 0.0001).

Our results showed that the STAT3 expression level increased in patients in comparison with the control group (P-value < 0.0001; Fig. 1a).

#### STAT3 methylation status in the study groups

Methylation level (% M) of the promoter region of STAT3 was present in 23% (12/50) of MS cases, while this level was found in 62.9% (45/50) of the controls. The decrease level was statistically significant (P-value < 0.0001). In addition, a significant and strong negative correlation was found between the STAT3 gene methylation level and mRNA expression level for the methylation assay (Figs. 1b–e).

### Discussion

FOXP3 is an essential transcription factor in the differentiation of Treg cells [40, 41]. Immunodysregulation, polyendocrinopathy, enteropathy, X-linked syndrome are disorders found in patients with FOXP3 mutations [22, 40]. FOXP3-deficient mice also exhibit eosinophilia, hyperimmunoglobulinemia E syndrome, and dysregulated production of Th1 and Th2 cytokines [42]. These observations verifies the major role of FOXP3 in Treg development, control immune tolerance, and homeostasis [15]. The CD28 costimulatory molecule is another factor required for the Treg development and peripheral conversion. Th17 cells have immunopathogenic potential, and their responses have been associated with the murine models of collagen-induced arthritis and experimental autoimmune encephalitis.

STAT3 is one of the regulating factors in the reciprocal development of Th17 cells and Tregs. A previous study has been shown that STAT3 is directly involved in the FOXP3 expression and Treg development [27]. In the present study, we observed STAT3 hypormethylation in RRMS patients and found that the STAT3 gene expression increases in RRMS patients, but not in the control subjects.

The results of the STAT3 methylation level and gene expression status in our study demonstrated the decreased level of methylation and the increased mean of mRNA expression in the patient group compared to the healthy one (hypomethylated). Therefore, our findings reveal a critical novel epigenetic event and new insights into the pathogenesis of MS disease. Regulation of the STAT3 in the present study maybe a novel promising treatment for MS as it has formerly been demonstrated that highly activated Th17 activity is related to STAT3 mutations [43]. Moreover, germline mutations in STAT3 causes the lymphoproliferation and early-onset autoimmunity [44]. An earlier investigation has reflected that STAT3-targeted therapeutics prevents experimental autoimmune uveitis mediated by Th17 cells [45]. STAT3 inhibitors are also effective in CNS autoimmune diseases [46].

Taken together, these findings affirm the role of STAT3 in Th17-mediated immune diseases. However, further studies are needed to fully elucidate the exact role of STAT3 in MS disease. STAT3 induction in the autoimmune therapy protocol is recommended.

## Limitations

The major concern of this study is the examination of STAT3 methylation on limited MS patients. The test of methylation on samples from various regions and in large areas in the country is suggested.

## Data Availability

The datasets used and/or analyzed during the current study are available from the corresponding author on reasonable request.

## Abbreviations

Treg: Regulatory T
STAT3: Signal transducer and activator of transcription 3
MS: Multiple sclerosis
CNS: Central nervous system
FOXP3: Forkhead box P3
Th17: T helper 17
IL: Interleukin
RRMS: Relapsing–remitting multiple sclerosis
NCBI: National Center for Biotechnology Information
GAPDH: Glyceraldehyde-3-phosphate dehydrogenase
SD: Standard deviation
DM: Demethylation
RT: Reverse transcriptase
qRT: Quantitative reverse transcriptase
SEM: Standard error of mean
